# A Case of Pla2r and Exostosin 1 Positive Membranous Nephropathy - The Diagnostic and Therapeutic Dilemma

**DOI:** 10.7759/cureus.43619

**Published:** 2023-08-17

**Authors:** Harish Sivagnanam, Murugesh Anand, PK Senthikumar, Kannan Bhaba Velu, Ramasubramanian Vishwanathan

**Affiliations:** 1 Nephrology, Tirunelveli Medical College, Tirunelveli, IND

**Keywords:** newer antigens for membranous nephropathy, membranous lupus, exostosin, pla2r, membranous nephropathy

## Abstract

Membranous nephropathy is an immune disease that commonly presents as nephrotic syndrome. The understanding of the pathogenesis of membranous nephropathy has rapidly evolved over the past few years due to the discovery of newer antigens. Exostosin 1 and exostosin 2 are antigens discovered in 2019 and found to be specific for membranous nephropathy secondary to autoimmune disease and are usually not seen in M-type phospholipase A2-associated membranous nephropathy. However, fewer clinical and pathological details of exostosin 1 and 2 related nephropathies are known, owing to the novelty of the antigen. Here we report a 24-year-old female who presented with nephrotic range proteinuria. Initial blood investigations revealed a doubtful autoimmune disease background. A subsequent renal biopsy revealed membranous nephropathy with both PLA2r and exostosin 1 positivity, which posed challenges in both diagnosis and treatment. Immunoglobulin G staining and electron microscopy were performed to differentiate if it was a PLA2r-associated or a exostosin 1/ exostosin 2-related membranous nephropathy. Electron microscopy revealed subepithelial deposits and immunoglobulin G stained for immunoglobulin G4, signifying possible PLA2r-associated membranous nephropathy with exostosin deposits. The patient was treated with rituximab and had a good treatment response. Only one similar case has been reported with both PLA2R and exostosin positivity. The pathophysiologic mechanism of this unique association is still unclear.

## Introduction

Membranous nephropathy (MN) is characterized by the subepithelial accumulation of immune complexes along the glomerular basement membranes. These immune complexes compromise IgG and its corresponding target antigen. Recent advances have led to the discovery of novel target antigens for MN. Approximately 90% of MN cases can now have target antigens identified [1]. MN accounts for nearly 20% to 37% of nephrotic syndrome cases in non-diabetic adults. The majority of MN cases are mediated by antibodies to the M-type phospholipase A2 receptor (anti-PLA2R) (70 to 80%) or thrombospondin type 1 domain containing 7A (THSD7A) (1%-5%) [2]. In 2019, exostosin 1 (EXT1) and exostosin 2 (EXT2) were identified as novel target antigens for MN secondary to autoimmune diseases [2]. Furthermore, exostosin positivity has been linked to young females with class V lupus and is typically associated with a favorable prognosis [3]. This case report highlights a unique instance of membranous nephropathy presenting with both PLA2R and EXT1 positivity, which posed both diagnostic and therapeutic challenges.

## Case presentation

The 24-year-old female was reportedly in good health until June 2021, when she experienced abdominal pain and burning during urination for a week. She was admitted to a private hospital for four days and treated for pyelonephritis. Throughout this period, there were no signs of reduced urine output, nausea, vomiting, facial puffiness, or leg swelling. Her renal function remained normal during this time (Table 1). Treatment included intravenous piperacillin and gentamicin for three days before being discharged.

**Table 1:** Investigations of our patient PCR - protein creatinine ratio, Hb - hemoglobin, Plt - platelet, TC - total count, IF - immunofluorescence, HIV - human immunodeficiency virus, HbsAg - hepatitis B surface antigen, anti-HCV - anti-hepatitis C virus, ELISA - enzyme-linked immunosorbent assay, ANCA - anti-neutrophil cytoplasmic antibody, PLA2R - anti-phospholipase A2 receptor, EXT1 - exostosin 1, NELL1 - neural epidermal growth factor-like protein 1

Investigations	Jun 2021	Oct 2021	Jul 2022	Nov 2022	Feb 2023	May 2023
Urine albumin	nil	4 +	3+	3+	2+	Trace
Urine PCR	NA	4.3	10	8	2.17	0.7
Hb g/dl	12.5	11.8	10.9	8.9	7.8	10.0
Tc cells/mm3	11800	9000	6700	10700	7300	7900
Plt lakh cells/mm3	2.6	2.52	2.32	4.32	2.93	4.2
Urea mg/dl	27	13	13	39	20	48
Creatinine mg/dl	0.8	0.6	0.7	1.6	1	1
Sodium mEq/l	NA	140	138	138	136	135
Potassium mEq/l	NA	4	4	3.6	4.2	3.6
Serum Albumin gm/dl	3.5	3	3	3.1	3.2	5
Lipid profile	NA	Total cholestorol: 366MG/DL	Total cholestorol: 312MG/DL			
Anti-nuclear antibody by IF	NA	NA	1 : 100 1 + spindle poles	Negative		Negative
C3 mg/dl (normal: 90 to 180 mg/dl)	NA	NA	212	261.45		
C4 mg/dl ( normal : 10- 40 mg/dl)	NA	NA	39	40.45		
Extended nuclear antigens	NA	NA	NA	Anti-AMA M2		
HIV	Negative	NA	Negative			
HbsAG	Negative	NA	Negative			
Anti-HCV	Negative	NA	Negative			
cANCA	NA	NA	NA	Negative		
pANCA	NA	NA	NA	Negative		
Serum anti-PLA2R RU/ml (normal < 14)	NA	NA	NA	34.34 RU/ml		9.34 RU/ml
Renal biopsy Immunofluorescence			IgG (3+), IgG4 +, C3 (2+) granular positivity in capillary loops			
Light microscopy			Spike formations in the glomerular basement membrane			
Tissue PLA2R				Diffuse granular capillary loop staining (3+)		
Tissue EXT1						Positive in the capillary loops
Tissue NELL1						Negative
Electron microscopy						Subepithelial electron-dense deposits. Thickened glomerular basement membrane diffuse effacement of the visceral epithelial cell foot processes

In October 2021, she developed facial puffiness over the course of a week and was subsequently diagnosed with nephrotic syndrome (Table 1). A renal biopsy was recommended, but she declined at that time and was lost to follow-up for about nine months. During this interval, she self-medicated with furosemide tablets. Her medical history did not include alopecia, photosensitivity, fever, oral ulcers, joint pain, swelling, skin rashes, hematuria, or native medication use. She reported experiencing oligomenorrhea and had been married since June 2022. There were no similar complaints within her family.

By July 2022, her symptoms had worsened, including the development of leg swelling and decreased urine output, leading to her hospitalization. Physical examination revealed pallor, bilateral pitting pedal edema, and periorbital edema. Her blood pressure measured 120/80 mm Hg. Immunofluorescence anti-nuclear antibody (ANA) testing showed a weak positive result of 1:100 titer with a spindle pole pattern. Elevated levels of C3 and C4 were detected. Kidney ultrasound demonstrated normal-sized kidneys with maintained corticomedullary differentiation.

**Figure 1:**
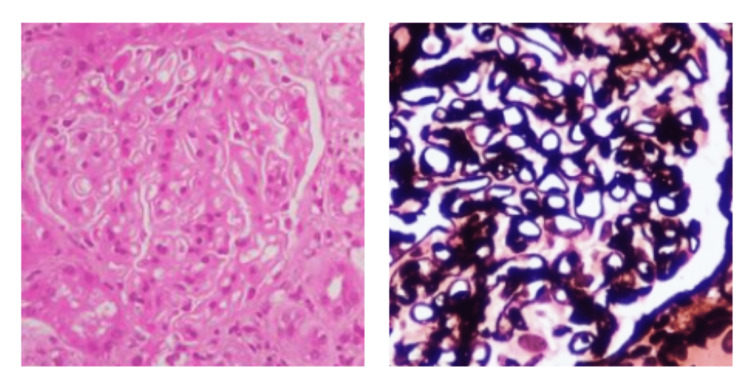
H&E stain (left) and silver stain (right) showing spike formation in the glomerular basement membrane (400x)

**Figure 2:**
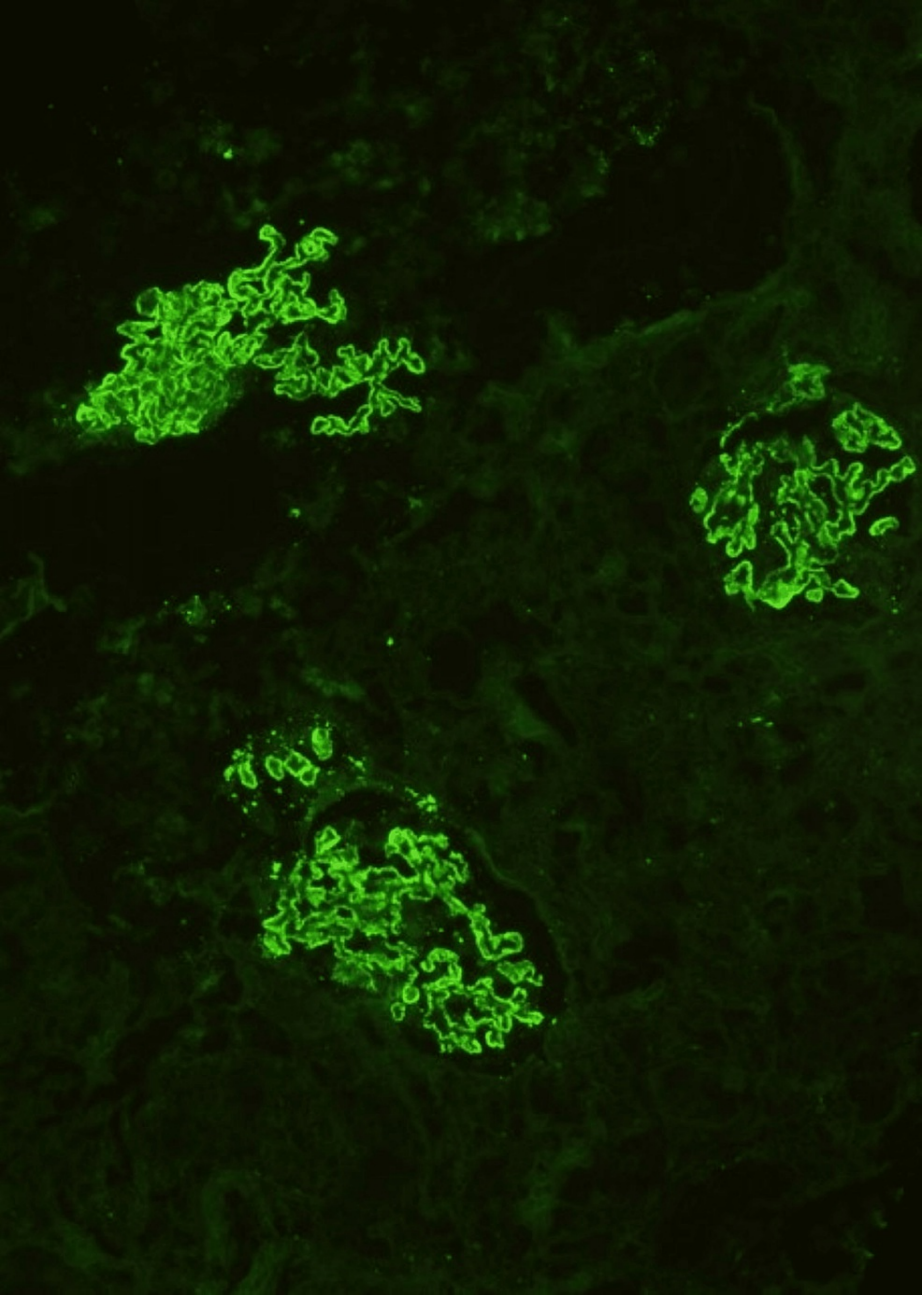
Immunofluorescence staining shows IgG granular positivity (3+) in the capillary loops (10x)

**Figure 3:**
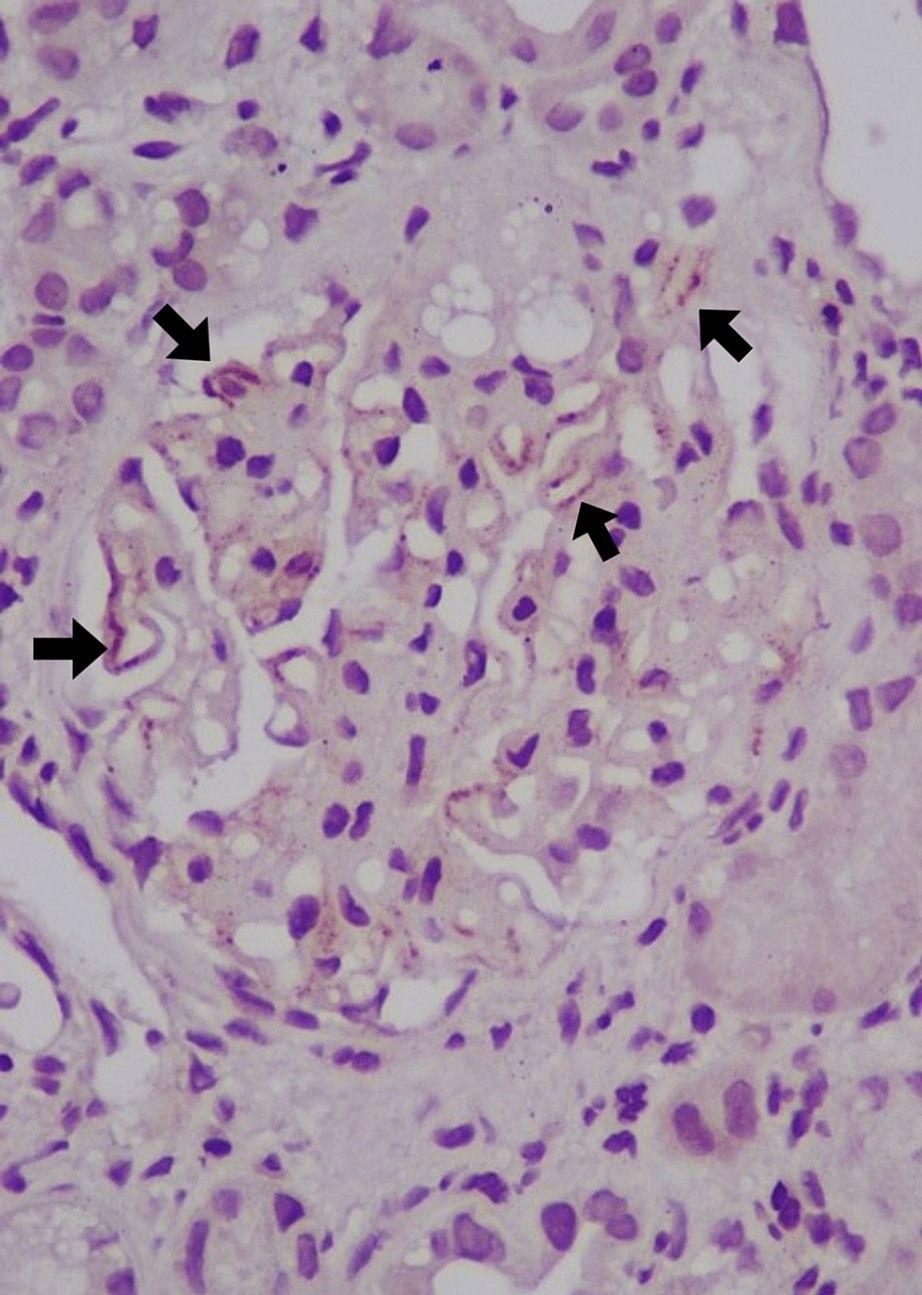
IgG4 staining shows focal positivity in the glomerular capillaries (IgG4 positivity denoted by black arrow, 400x)

A renal biopsy was performed, revealing 13 glomeruli with spike formations evident in the glomerular basement membrane. Notably, there were no signs of segmental sclerosis, endocapillary hypercellularity, crescents, interstitial fibrosis, tubular atrophy, or inflammatory infiltrate (Figure 1). Immunofluorescence demonstrated granular positivity for IgG (+3) and C3 (+2) in the capillary loops, while subclass staining for IgG4 was also positive in the capillary loops (Figures 2, 3). Further details can be found in Table 1. The patient was initiated on enalapril 2.5 mg twice daily and maintained regular follow-ups, including discussions about the potential pregnancy risks associated with angiotensin-converting enzyme (ACE) inhibitors.

**Figure 4:**
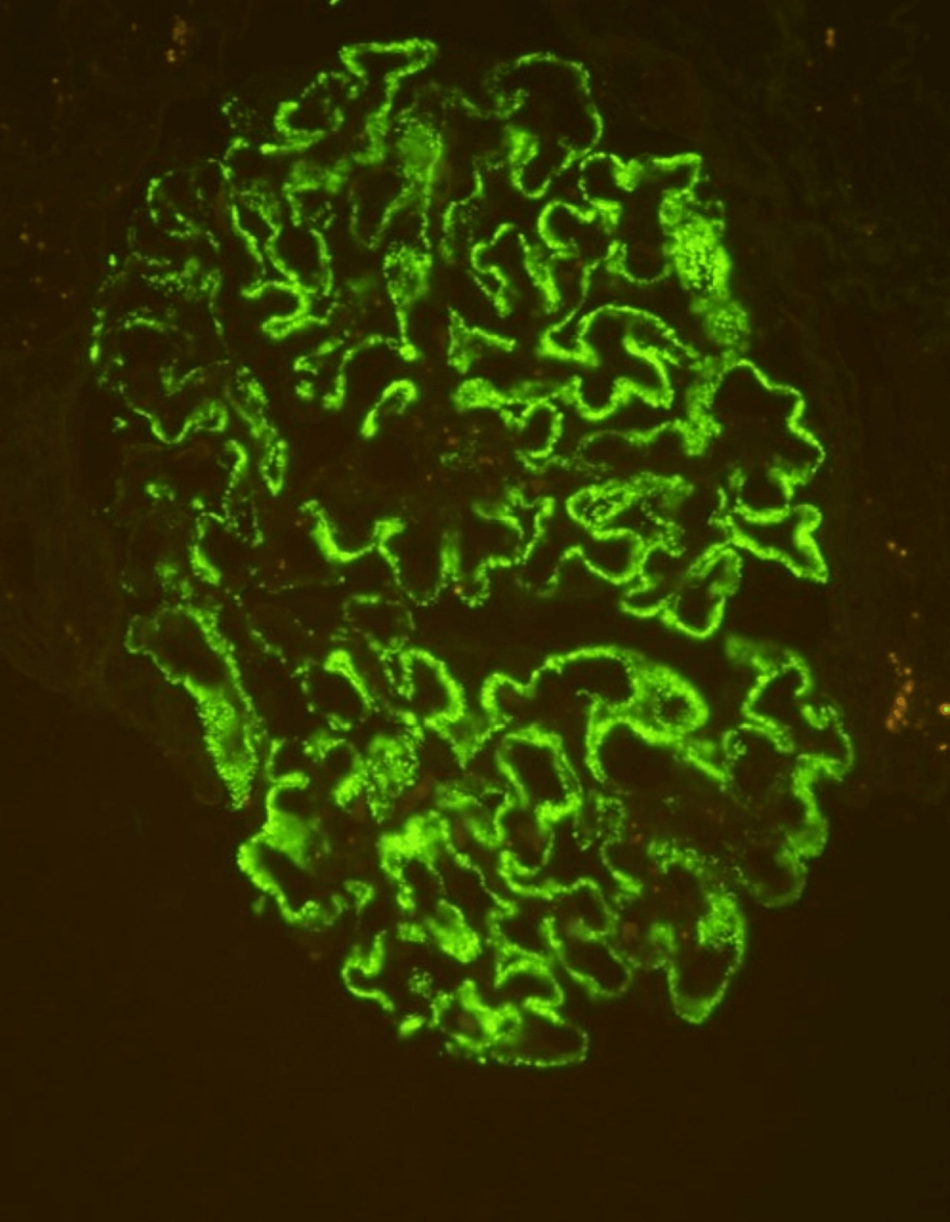
Glomeruli PLA2R shows diffuse, granular, capillary loop staining (3+), 400x PLA2R - phospholipase A2 receptor antibody

In November 2022, her symptoms worsened, prompting a visit to a private hospital. ANA testing was repeated and yielded a negative result, while extended nuclear antigens revealed anti-mitochondrial antibody (AMA M2) positivity. There was no evidence of auto-immune hepatitis and primary biliary cirrhosis (PBC). As a result, she commenced treatment with prednisolone 20 mg. However, she was readmitted to our hospital two weeks after initiating steroid therapy due to left lower limb cellulitis. Antibiotics were administered, and she underwent reevaluation. Repeat ANA testing again returned a negative result, alongside normal C3 and C4 levels. Both antineutrophil cytoplasmic antibodies (cANCA and pANCA) were negative. Given the negative ANA result, serum anti-PLA2r antibodies were assessed, and tissue PLA2r exhibited diffuse granular capillary loop staining at a 3+ intensity (Figure 4). Concurrently, her serum anti-PLA2r levels were elevated. Notably, her urine protein creatinine ratio (PCR) was measured at 10.0 during this period.

She was presented with all available treatment options for Membranous nephropathy, including cyclophosphamide, rituximab, and calcineurin inhibitors (CNIs). Opting for rituximab, she commenced treatment with rituximab 1g IV in December 2022 following the resolution of her cellulitis. Irregular follow-up was noted, and a second dose of rituximab was administered in February 2023. During this period, her investigative results are detailed in Table 1. In April 2023, she underwent an abortion at six weeks of pregnancy. In May 2023, she attended a routine check-up, during which a urine examination indicated a urine PCR level of 3.0.

**Figure 5:**
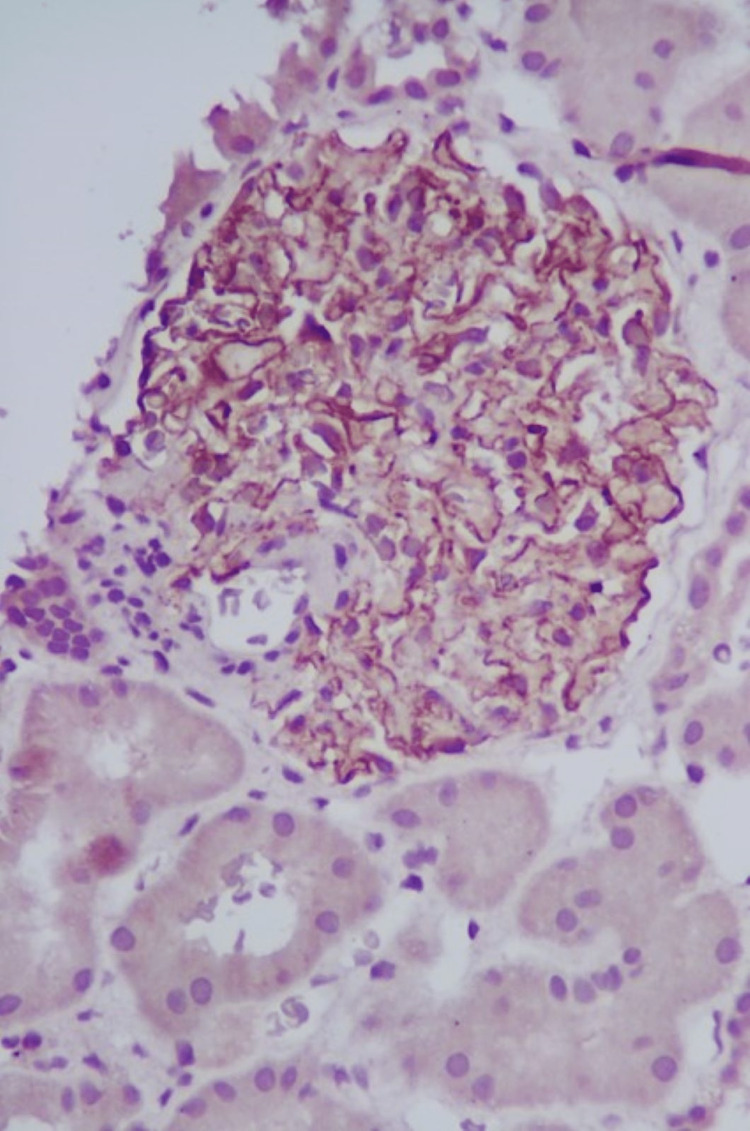
Exostosin 1 stains positivity on the glomerular capillaries (400x)

**Figure 6:**
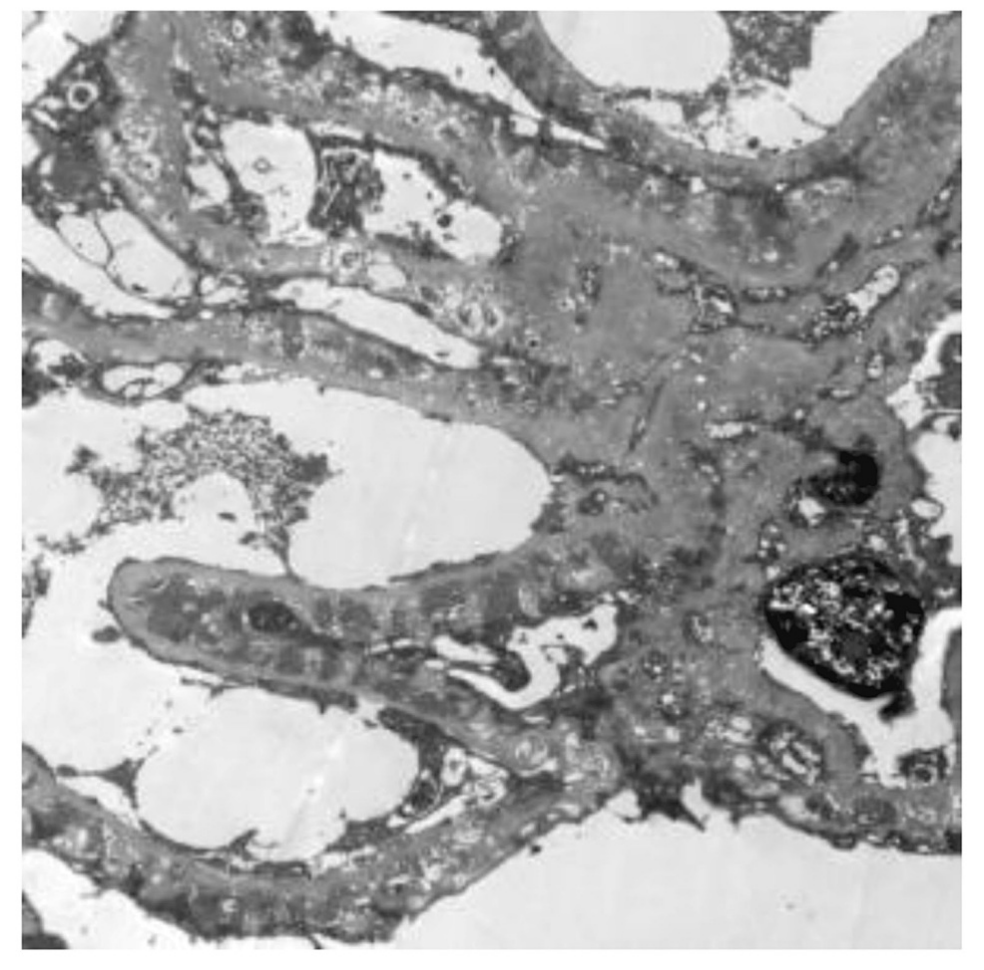
Electron microscopy of glomerulus showing multiple subepithelial deposits, foot process effacement, and glomerular basement membrane thickening

Upon reconsideration of her medical records, prompted by persistent proteinuria and to rule out potential membranous lupus, EXT1 staining was performed, revealing positive staining in the capillaries (Figure 5). However, NELL1 staining yielded negative results. Notably, electron microscopy unveiled multiple sub-epithelial electron-dense deposits, a thickened glomerular basement membrane, and diffuse foot process effacement of the visceral epithelial cells (Figure 6).

The patient's condition has demonstrated symptomatic improvement, prompting regular follow-up. Her ongoing management includes ACE inhibitors, and her current urine PCR level stands at 0.7. Moreover, a repeat serum PLA2r measurement indicated a low level of 9.34 RU/ml.

## Discussion

Membranous nephropathy is a prevalent cause of nephrotic syndrome in adults. As of 2023, target antigens can be identified in 90% of cases of membranous nephropathy. The antigens identified include phospholipase A2 receptor (PLA2r), thrombospondin type 1 domain-containing 7A (THSD7A), exostosin 1 (EXT1) and exostosin 2 (EXT2), neural epidermal growth factor-like 1 (NELL1), neutral endopeptidase (NEP), semaphorin 3B (SEMA3B), neural cell adhesion molecule 1 (NCAM1), neuron-derived neurotrophic factor (NDNF), fat cadherin 1 (FAT1), proprotein convertase subtilisin/kexin type 9 (PCSK9), etc. [1].

The management of PLA2r-positive MN primarily relies on the modified Ponticelli regimen, rituximab, and calcineurin inhibitors (CNIs). However, membranous lupus is treated with cyclophosphamide (NIH or EuroLupus) and mycophenolate mofetil (MMF) [4]. Thus, distinguishing between these two diseases is crucial. In the original study by Sethi et al., 47 PLA2r-positive MN samples (including the pilot and discovery cohorts) were examined for Exostosin 1 and 2 (EXT1/EXT2), and all tested negative for EXT1/EXT2 [2]. Subsequently, EXT1/EXT2-associated MN has been linked exclusively to younger females with autoimmune diseases. EXT1/ EXT2-associated MN was found to be associated with a good prognosis [3]. Limited reports exist on EXT1-associated nephropathy. Notably, our case exhibited no clinical features of an autoimmune disorder. Although initial anti-nuclear antibody (ANA) testing yielded positive results (1:100, spindle poles pattern), consecutive ANA tests were consistently negative. Extended nuclear antigen tests identified anti-AMA M2 antibodies without any features of autoimmune hepatitis or primary biliary cirrhosis. Electron microscopy revealed classic features of PLA2r-associated MN, including subepithelial deposits, foot process effacement, and glomerular basement membrane thickening [5].

This case presents a unique scenario of PLA2r-associated MN with EXT1 positivity, unreported in original or follow-up studies [2,3]. Nevertheless, a single case of PLA2r-positive membranous nephropathy with EXT1/EXT2 positivity has been documented, with only one out of 39 PLA2r-associated MN samples testing positive for EXT1/ EXT2 [6]. A study by Iwakura et al. suggested that EXT1/EXT2-associated MN may occur independently of autoimmune diseases, proposing it as a potential marker for autoimmune disease occurrences in the future [7]. Pathophysiologic mechanisms of exostosin deposits in PLA2r-associated MN are still unclear. Additionally, PLA2r positivity has been observed in membranous lupus cases, with worse remission rates and prolonged remission duration associated with PLA2r positivity [8]. Another retrospective Indian study revealed 13.5% of secondary MN cases exhibiting PLA2r positivity [9]. In this case, considering the electron microscopy features, which showed only sub-epithelial deposits (vs. subendothelial and mesangial deposits in membranous lupus), IgG4 deposits in the glomerular capillaries and good response to rituximab IV, we made a diagnosis of PLA2r associated MN with exostosin deposits [5]. The patient is in remission and on regular monthly follow up. As newer antigens reshape our understanding of membranous nephropathy, the primary diagnostic challenge, in this case, is differentiating between PLA2r-associated MN with EXT1 deposits or EXT1-associated MN with PLA2R positivity. Although electron microscopy, IgG subtyping, and distinct treatment modalities offer insights, their limited routine use in India underscores the importance of reporting such unique cases to advance EXT1/ EXT2-associated MN research.

## Conclusions

In summary, this intricate case of membranous nephropathy, characterized by the presence of PLA2r and EXT1 positivity, underscores the dynamic nature of glomerular disorders. The pathophysiological mechanism of exostosin deposition in PLA2r-associated membranous nephropathy is still unclear. The diagnostic complexities, therapeutic choices, and unforeseen antigen correlations, in this case, underscore the imperative for continuous research regarding antigens in membranous nephropathy. As we progressively unravel the nuances of membranous nephropathy, our comprehension of these conditions will deepen, facilitating the development of refined diagnostics and individualized treatments in the forthcoming era.
